# Altered gut microbiota in Taiwanese A97S predominant transthyretin amyloidosis with polyneuropathy

**DOI:** 10.1038/s41598-024-56984-5

**Published:** 2024-03-14

**Authors:** Chieh-Chang Chen, Ping-Huei Tseng, Hsueh-Wen Hsueh, Ming-Chang Chiang, Shiou-Ru Tzeng, Tsung Hsien Chiang, Ming-Shiang Wu, Sung-Tsang Hsieh, Chi-Chao Chao

**Affiliations:** 1 Departments of Gastroenterology, National Taiwan University Hospital, Taipei, Taiwan; 2 Departments of Neurology, National Taiwan University Hospital, Taipei, Taiwan; 3 Department of Biomedical Engineering, National Yang Ming Chiao Tung University, Taipei, Taiwan; 4 Institute of Biochemistry and Molecular Biology, College of Medicine, National Taiwan University, Taipei, Taiwan; 5 Department of Anatomy and Cell Biology, College of Medicine, National Taiwan University, Taipei, Taiwan

**Keywords:** Neuroscience, Gastroenterology, Neurology

## Abstract

Increasing evidence suggests that gut microbiota alterations are related to development and phenotypes of many neuropsychiatric diseases. Here, we evaluated the fecal microbiota and its clinical correlates in patients with hereditary transthyretin amyloidosis (ATTRv) and polyneuropathy. Fecal microbiota from 38 ATTRv patients and 39 age-matched controls was analyzed by sequencing 16S V3–V4 ribosomal RNA, and its relationships with clinical characteristics of polyneuropathy and cardiomyopathy were explored. The familial amyloidotic polyneuropathy stage was stage I, II, and III in 13, 18, and 7 patients. ^99m^Tc-PYP SPECT showed a visual score of 2 in 15 and 3 in 21 patients. The gut microbiota of ATTRv patients showed higher alpha diversity (ASV richness and Shannon effective numbers) and dissimilar beta diversity compared to controls. Relative abundance of microbiota was dominated by Firmicutes and decreased in Bacteroidetes in ATTRv patients than in controls. Patients with more myocardial amyloid deposition were associated with increased alpha diversity, and the abundance of Clostridia was significantly correlated with pathophysiology of polyneuropathy in ATTRv patients. These findings demonstrated alterations in the gut microbiota, especially Firmicutes, in ATTRv. The association between altered microbiota and phenotypes of cardiomyopathy and polyneuropathy might suggest potential contributions of gut microbiota to ATTRv pathogenesis.

## Introduction

Hereditary transthyretin (TTR) amyloidosis (ATTRv) is an inherited peripheral neurodegenerative disease caused by mutations in the TTR gene. Mutated TTR can cause amyloid deposition in various tissues, especially the peripheral nervous system and heart, and patients will gradually develop extensive neuropathy and cardiomyopathy^1^. The epidemiological features and clinical manifestations of ATTRv are variable, even in patients with the same gene mutations. For example, ATTRv due to the V30M mutant, the most common genotype worldwide, can manifest as early- or late-onset disease. A97S mutant, the most common gene mutation in Taiwan and Southeast Asia^2–5^, usually presents as a late-onset disease, however, the individual onset age varies greatly, ranging from 40 to 75 years old, and the penetrance of this mutant is incomplete^2,6,7^. Additionally, amyloidosis may primarily affect the peripheral nervous system in some patients and the heart in others^8^; the involvement of sensory, motor or autonomic nerves among patients varies, and the sex distribution is quite unequal^6,9,10^. It is unclear why ATTRv has such great differences in its manifestations, and unknown factors are suspected to influence the phenotypes of ATTRv.

The gut microbiota refers to the many bacterial groups inhabiting the human intestine and is related to the decomposition of food, nutrient processing, and immune responses; furthermore, the gut microbiota plays a role in various physiological functions and pathological changes in organs, including the nervous system^11^. There is a two-way signal transmission between the gastrointestinal tract and the brain, and increasing evidence suggests that such bidirectional communication between the enteric and central nervous systems is affected by the gut microbiota^12^. Many studies have documented the link between changes in the gut microbiota and neuropsychiatric disorders, including Parkinson’s disease, autism, and Alzheimer's disease, and the phenotypes of such disorders are associated with alterations in the gut microbiota^13–15^.

The peripheral nervous system is closely connected and interacts with the central nervous system, and interactions between the brain and gut as well as its microbiota rely on peripheral nerves. It is possible that not only the brain but also the peripheral nervous system may be affected by the gut microbiota. Significant alteration of gut microbiota has been found in diabetic distal symmetric polyneuropathy and chronic inflammatory demyelinating polyneuropathy^16,17^. Gastrointestinal dysfunction, such as constipation, diarrhea and bile acid malabsorption resulted from autonomic neuropathy or amyloid depositions along the intestine, is frequently seen in ATTRv and may be associated with dysbiosis of the gut microbiota^18–20^. However, the alteration of the gut microbiota in ATTRv has not been well explored before. In the present study, we aimed to characterize the fecal microbiota in a Taiwanese ATTRv population with predominant A97S mutation, and analyzed the relationship between the fecal microbiota and the clinical phenotypes to investigate whether the gut microbiota could contribute to the pathogenesis of ATTRv.

## Results

### Clinical profiles of ATTRv patients

Thirty-eight patients (29 men), aged 63.7 ± 6.0 years (range 52–78 years), with confirmed mutations in the TTR gene and ATTRv were enrolled. The TTR mutations were p.Ala117Ser (A97S) in 36 patients, p.Phe53Leu (F33L) in 1 patient, and p.Glu109Lys (E89K) in 1 patient. The onset age of polyneuropathy was 60.1 ± 5.7 years (range 50–70.0 years), and the disease duration at the time of study was 3.5 ± 2.0 years. None of these patients had major medical comorbidities such as diabetes mellitus, hypertension or uremia. At enrollment, 17 patients were treatment-naive and 21 patients received diflunisal treatment. All patients had sensory symptoms in the distal limbs, and 35 patients (92.1%) manifested limb weakness. Twenty-six (68.4%) patients experienced neuropathic pain. Thirty-five patients (92.1%) suffered from autonomic impairment, including gastrointestinal dysfunction (34 patients), orthostatic hypotension (21), sudomotor dysfunction (28) or genitourinary dysfunction (24). For gastrointestinal dysfunction, constipation and/or diarrhea were noted in 32 patients. In patients receiving 99mTc-PYP SPECT imaging (n = 36), all showed significant radiotracer uptake in the heart, and the visual score was grade 2 in 15 patients (41.7%) and grade 3 in 21 patients (58.3%). According to the clinical severity of polyneuropathy, 13 patients who ambulated without assistance were scored as FAP stage 1, 18 patients needing unilateral or bilateral assistance for walking were scored as stage 2, and 7 patients dependent on wheelchairs for ambulation were scored as stage 3 (Table 1). In the WHOQoL, the score for overall health was 2.21 ± 0.55, and the scores were 12.70 ± 2.43 for the physical domain, 14.58 ± 1.87 for the psychological domain, 15.42 ± 1.62 for the social relationship domain, and 15.48 ± 1.15 for the environment domain.

**Table 1:** Demographic and clinical characteristics of hereditary transthyretin amyloidosis (ATTRv) patients and controls.

	ATTRv patients	Controls
Number of patients	38	39
Age of onset	60.1 ± 5.7 years	–
Age at enrollment	63.7 ± 6.0 years	61.7 ± 10.2 years
Sex (Female:Male)	9:29	29:10
Genotypes	p.Ala117Ser (A97S), n = 36	–
	p.Phe53Leu (F33L), n = 1	
	p.Glu109Lys (E89K), n = 1	
FAP stage	FAP stage 1, n = 13	–
	FAP stage 2, n = 18	
	FAP stage 3, n = 7	
Treatment	No treatment, n = 17	–
	Diflunisal, n = 21	
99mTc-PYP SPECT	VS2, n = 15	–
	VS3, n = 21	
Symptoms at enrollment		
Motor weakness	n = 35, 92.1%	n = 0
Sensory deficits	n = 38, 100%	n = 0
Autonomic dysfunction	n = 35, 92.1%	n = 0
Diarrhea/constipation	n = 32, 84.2%	n = 0
Neuropathic pain	n = 26, 68.4%	n = 0

FAP stage, familial amyloidotic polyneuropathy stage; n, number of patients; VS, visual score; 99mTc-PYP, 99 m-technetium pyrophosphate.

To compare the gut microbiome changes, we recruited 39 unrelated family members (10 men), aged 61.7 ± 10.2 years (range 27–85 years), who lived with the enrolled ATTRv patients as a control group, and all of them were the spouses of patients except two: one was a care giver (27 years old) and the other was the son-in law (39 years old) of the patients (Table 1). There was no difference in age (*p* = 0.296, Supplementary Fig. 1) but a significant difference in sex (*p* < 0.001) between ATTRv patients and controls.

### Neuropathy profiles in ATTRv patients

Table 2 summarized the data of the nerve conduction studies, intraepidermal nerve fiber density analyses and quantitative sensory tests. All patients showed features of polyneuropathy in nerve conduction studies. The IENF density was 1.75 ± 1.98 fibers/mm, and approximately four-fifths (81.6%) of patients had abnormally reduced IENF density. For quantitative sensory testing, 47.4–71.1% of patients had abnormally elevated thermal thresholds at the hands and feet, and 57.9% and 86.8% of patients had elevated vibratory thresholds at the index finger and ankle, respectively.

**Table 2:** Skin innervation, neurophysiological, and psychophysiological data of patients with hereditary transthyretin amyloidosis (ATTRv).

Large fiber nerves	n = 38 Value (Abnormal rate)
Nerve conduction study	
Median nerve	
Distal motor latency (ms)	6.4 ± 1.9 (91.9%)
Distal CMAP (mV)	2.2 ± 2.0 (89.2%)
Motor NCV (m/s)	44.7 ± 6.9 (81.1%)
Distal SNAP (μV)	2.9 ± 7.5 (91.9%)
Sensory NCV (m/s)	44.0 ± 10.3 (94.7%)
Ulnar nerve	
Distal motor latency (ms)	3.8 ± 0.9 (55.3%)
Distal CMAP (mV)	4.5 ± 3.0 (65.8%)
Motor NCV (m/s)	49.4 ± 6.4 (47.4%)
Distal SNAP (μV)	5.6 ± 7.3 (86.8%)
Sensory NCV (m/s)	50.7 ± 6.8 (57.9%)
Peroneal nerve	
Distal motor latency (ms)	5.0 ± 1.4 (52.6%)
Distal CMAP (mV)	1.1 ± 1.8 (79.0%)
Motor NCV (m/s)	39.2 ± 5.7 (76.3%)
Tibial nerve	
Distal motor latency (ms)	4.6 ± 1.5 (37.8%)
Distal CMAP (mV)	2.1 ± 4.2 (89.2%)
Motor NCV (m/s)	37.6 ± 6.7 (75.7%)
Sural nerve	
Distal SNAP (μV)	1.4 ± 3.2 (84.2%)
Sensory NCV (m/s)	44.9 ± 8.2 (84.2%)
Quantitative sensory testing	
Vibratory at index finger	20.8 ± 38.8 (57.9%)
Vibratory at ankle	62.8 ± 51.6 (86.8%)

Small fiber nerves	n = 38 Value (Abnormal rate)
Skin biopsy	
Intraepidermal nerve fiber density (fibers/mm)	1.75 ± 1.98 (81.6%)
Quantitative sensory testing	
Warm at thenar (°C)	38.3 ± 6.2 (68.4%)
Cold at thenar (°C)	24.2 ± 11.0 (65.8%)
Warm at foot dorsum (°C)	44.3 ± 4.8 (71.1%)
Cold at foot dorsum (°C)	19.9 ± 11.9 (47.4%)

CMAP, compound muscle action potential; NCV, nerve conduction velocity; n, number of patients; SNAP, sensory nerve action potential.

### Altered and more diverse gut microbiota in ATTRv patients

**Figure 1:**
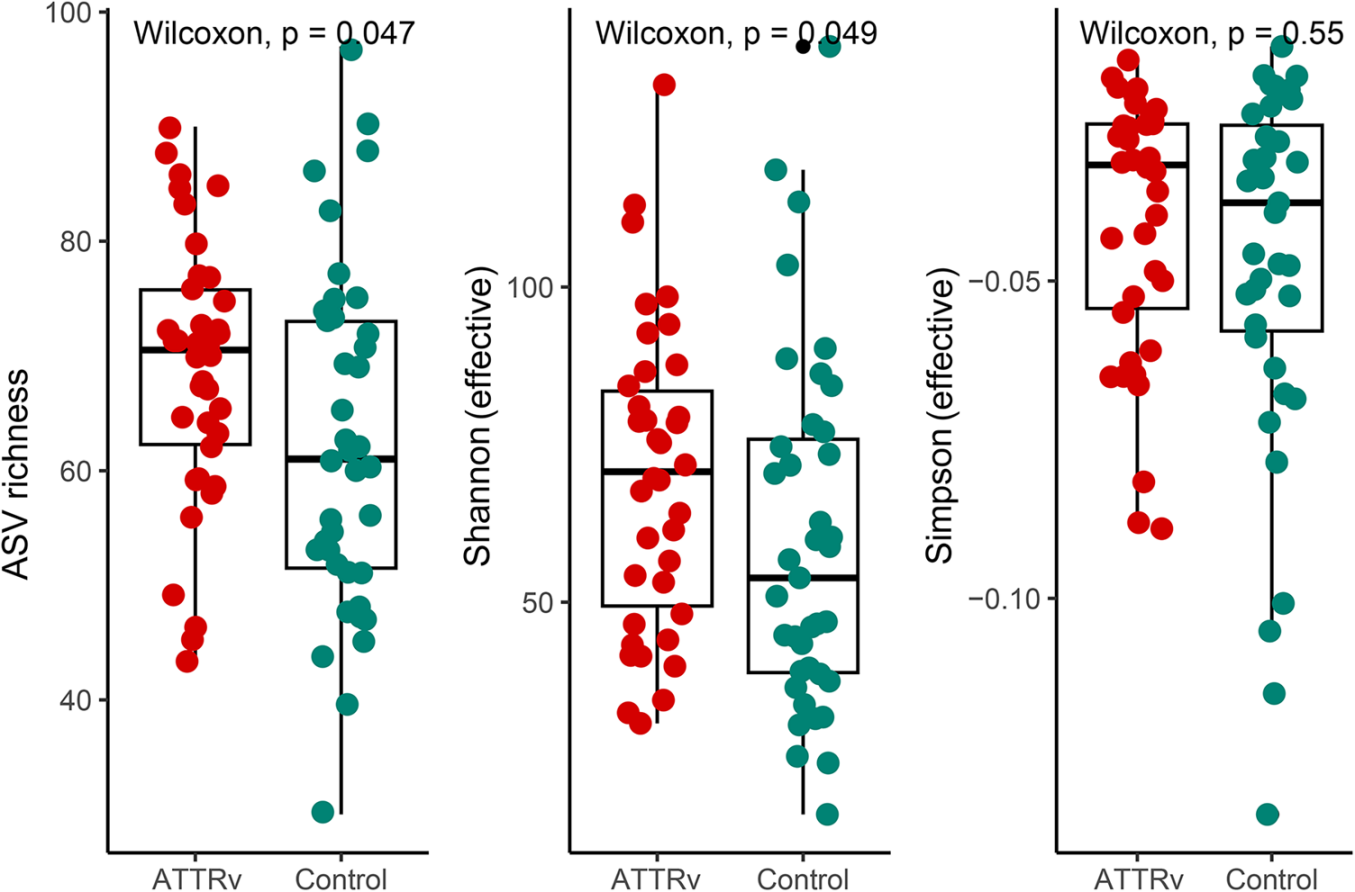
The alpha diversity of gut microbiota in patients with hereditary transthyretin amyloidosis (ATTRv) and the control group. The effective numbers of species in gut microbiota expressed by amplicon sequence variants (ASV) richness and Shannon effective numbers were higher in patients with ATTRv patients than in the control group.

**Figure 2:**
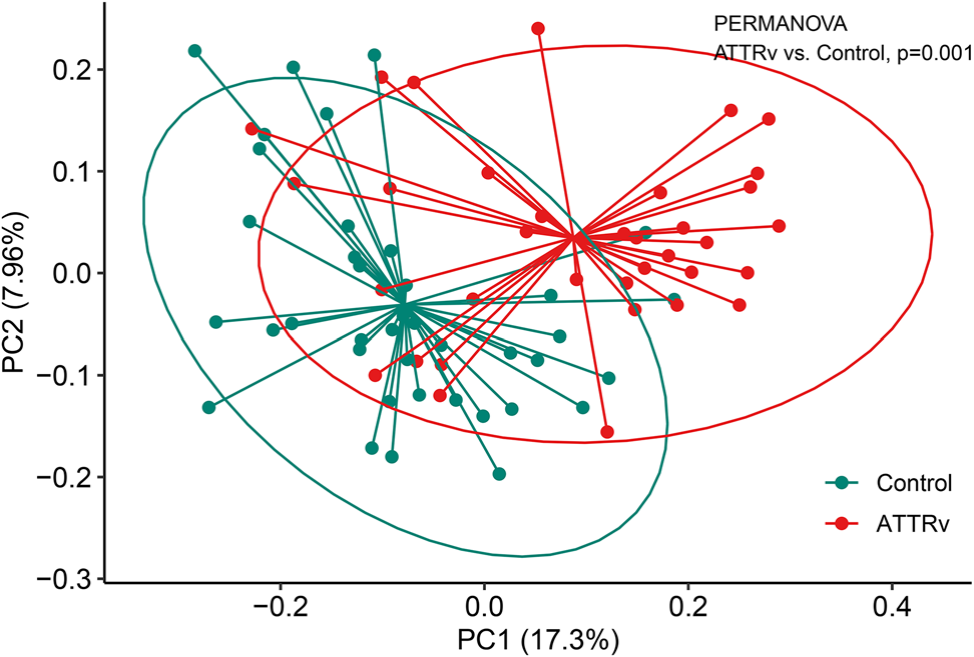
Beta diversity analysis of gut microbiota in patients with hereditary transthyretin amyloidosis (ATTRv) and the control group. The plot of principal coordinate analysis based on Bray‒Curtis dissimilarity showed a significant difference in the gut microbiota between the groups.

**Figure 3:**
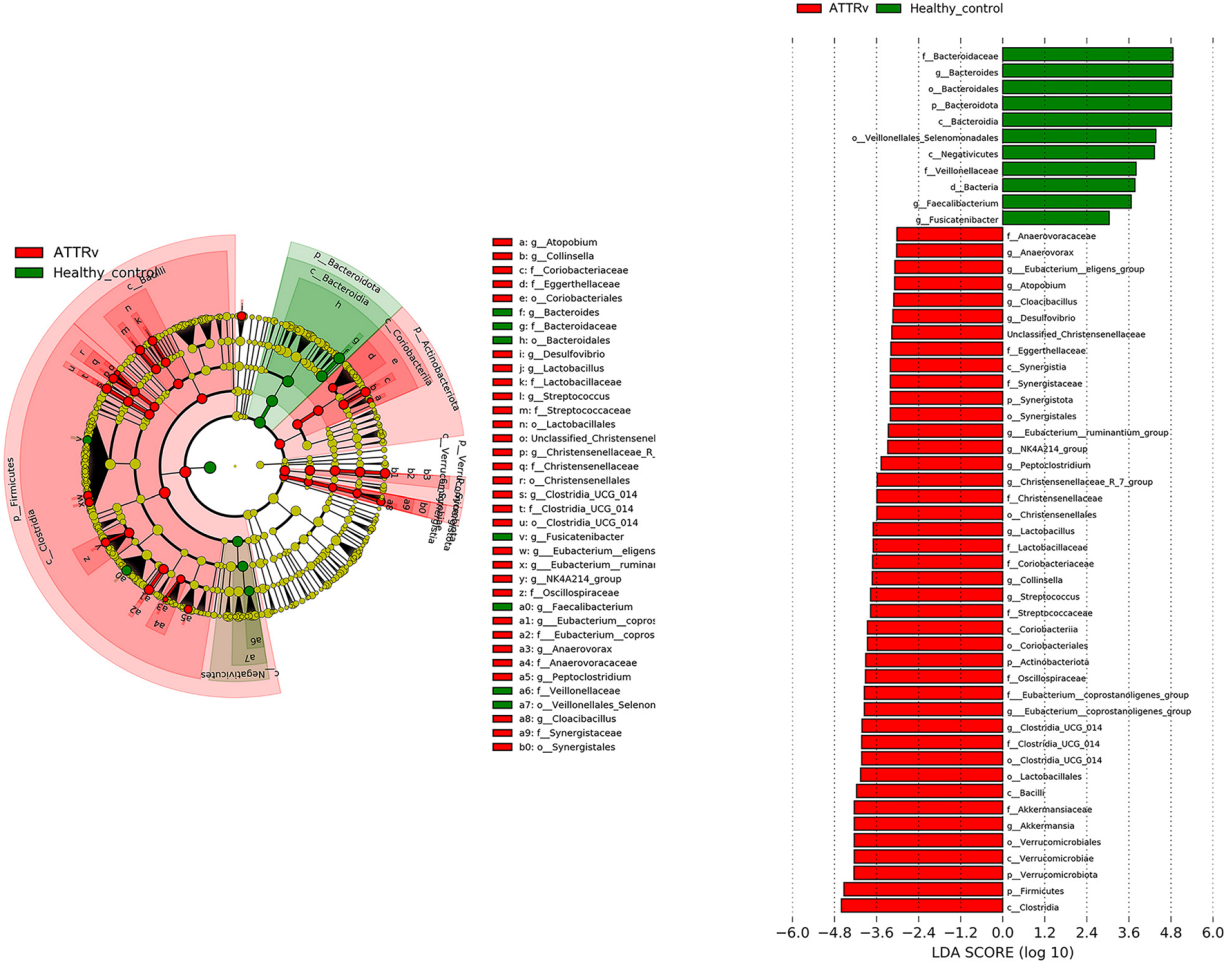
Taxonomic differences in gut microbiota in patients with hereditary transthyretin amyloidosis (ATTRv) and the control group. The cladogram (left) using the LEfSe method demonstrated the phylogenetic distribution of gut microbiota associated with patients with ATTRv (red) and control subjects (green). Linear discriminant analysis scores (right) showed a significant bacterial difference between the ATTRv patients and the control group.

We then investigated changes in the microbiota of the gut in ATTRv patients by high-throughput sequencing of the V3–V4 region of the 16S ribosomal RNA gene. We evaluated the amplicon sequence variants (ASV) richness and diversity within each sample from both ATTRv patients and the control group by different metrics: the ASV richness, Shannon effective numbers and Simpson effective numbers. There were statistical differences in the ASV richness (*p* = 0.046) and Shannon effective numbers (*p* = 0.049), suggesting a higher abundance of gut microbiota in ATTRv patients (Fig. 1). To compare the difference in the composition of the gut microbiota between ATTRv patients and controls, we next explored the beta diversity of the samples by generalized UniFrac distances. PCoA revealed significant differences in gut microbiota between ATTRv patients and controls (*p* = 0.001 by PERMANOVA), suggesting significant dissimilarity of the gut microbiota between ATTRv patients and controls (Fig. 2). We further compared the differences in the microbiota between the ATTRv and control groups by linear discriminant analysis (LDA) effect size (LEfSe) to determine the presence and effect size of region-specific genera. A logarithmic LDA score cutoff of 3.0 was applied to identify important taxonomic differences between the ATTRv and control groups. Figure 3 shows the difference in fecal microbiota between the FAP and control groups based on LEfSe analysis and a cladogram. There was a decrease in the relative abundances of Bacteroidetes, Veillonelales and Selenomonadales in the ATTRv group compared to the control group, while the relative abundances of Clostridia, Akkermansia, Eubacteria, Streptococcus, Lactobacillus, Christensenellaceae, Synergistaceae, Coriobacteriaceae, Eggerthellaceae, and Anaerovoracaceae were higher in ATTRv patients than in controls. Since many of the above microbiota with significant changes in abundance belong to Firmicutes and Bacteroidetes, which are the two dominant bacterial phyla in the human gut, we then analyzed the Firmicutes to Bacteroidetes ratio, which is widely accepted to have an important influence in maintaining normal intestinal homeostasis. This ratio was much higher in ATTRv patients than in controls (13 [0, 1950] vs. 2 [0, 1354], median [IQR], *p* < 0.001 by Mann‒Whitney U test).

For the possible interference of treatment and gastrointestinal dysfunction like diarrhea and constipation on the richness and compositions of gut microbiota, we examined the alpha- and beta-diversity between ATTRv patients with and without treatment, diarrhea or constipation. There was no difference in alpha and beta diversity between ATTRv patients with and without treatment, diarrhea or constipation (Supplementary Fig. 1).

### Clinical correlates of fecal microbiota in ATTRv

**Figure 4:**
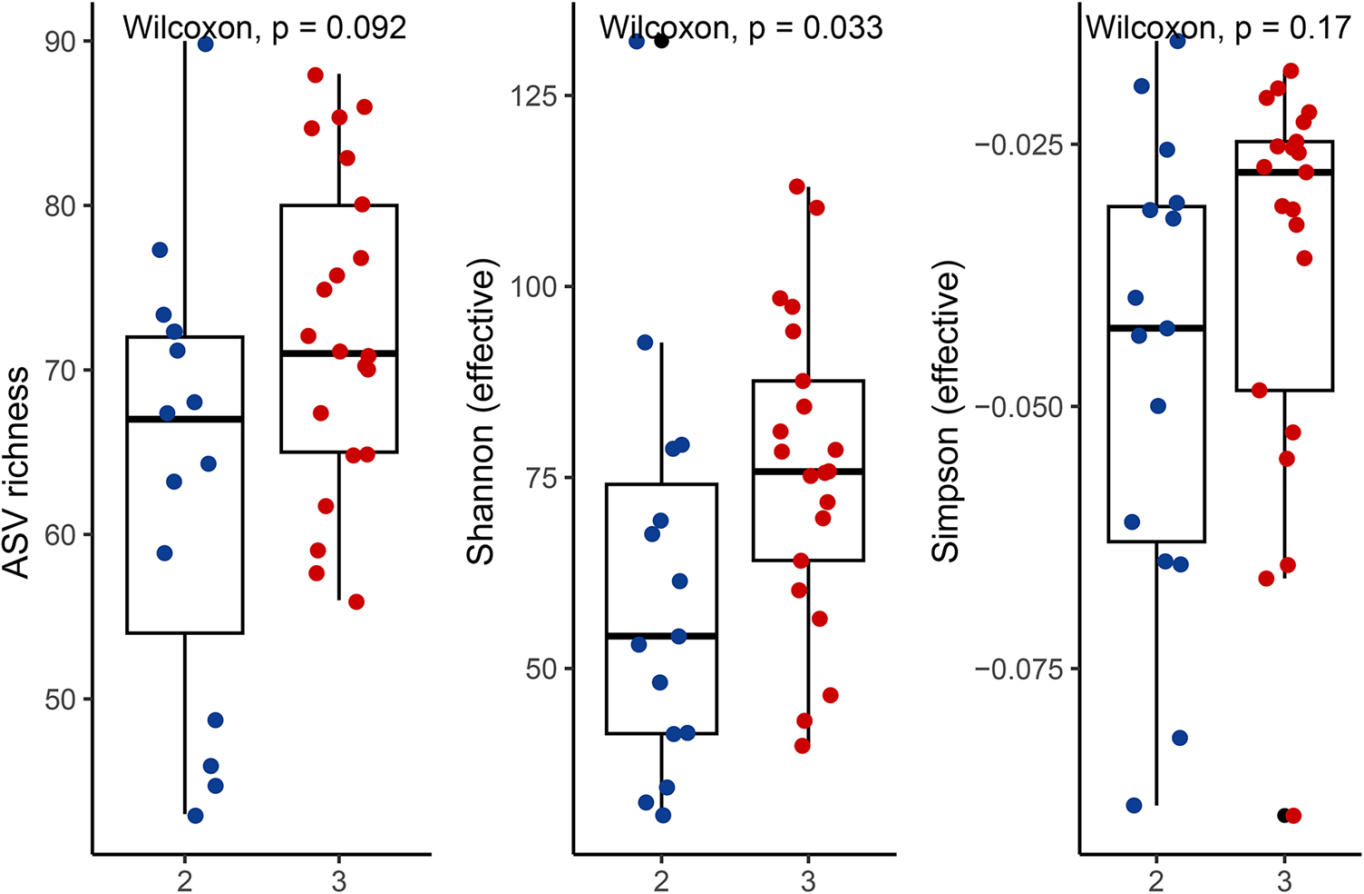
The clinical significance of alpha diversity of gut microbiota in patients with hereditary transthyretin amyloidosis (ATTRv). The alpha diversity measured by Shannon effective numbers was higher in patients with a visual score of grade 3 on 99mTc-PYP SPECT imaging than in those with a visual score of grade 2.

**Figure 5:**
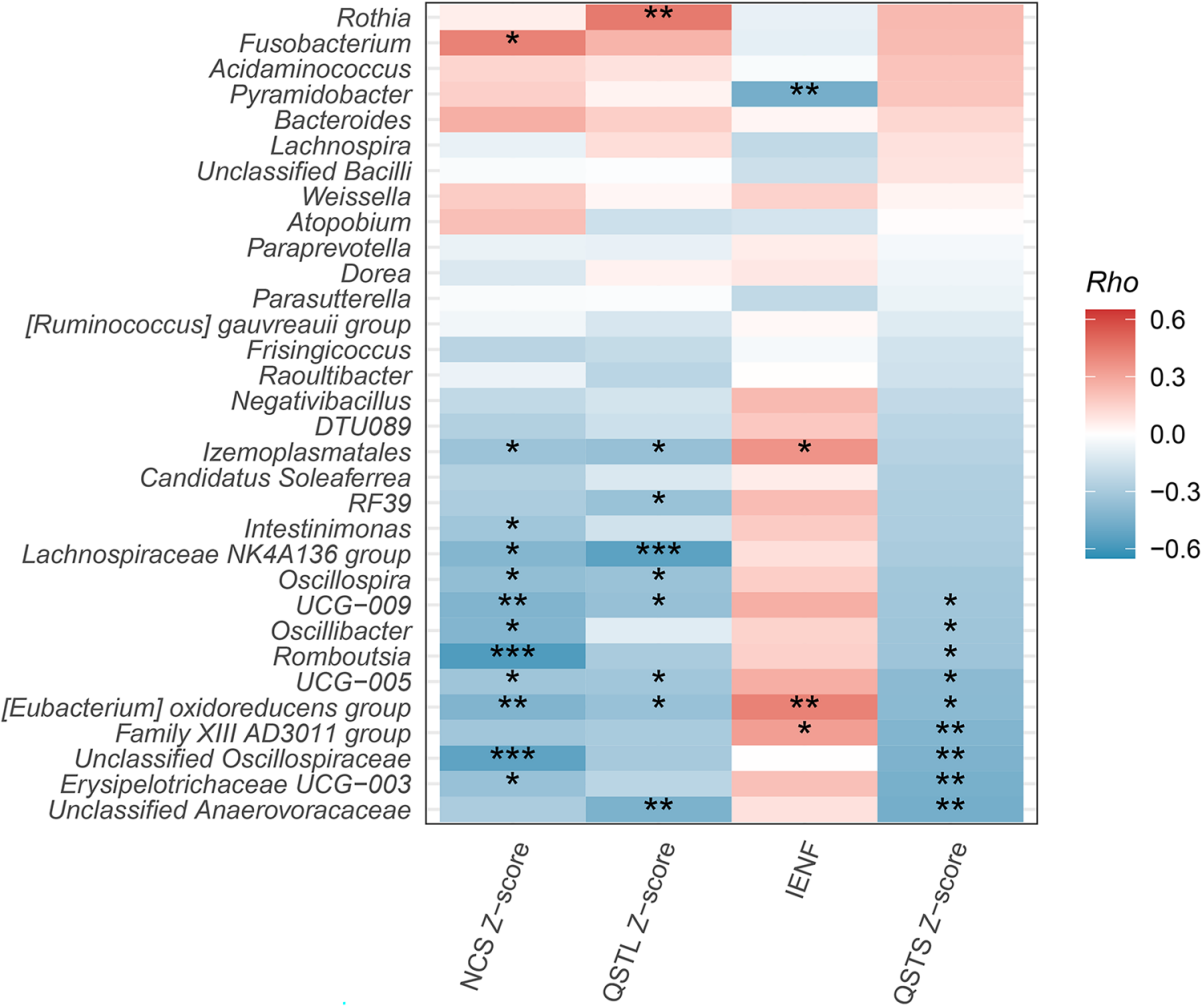
The correlation heatmap of the gut microbiota with the neuropathic parameters of patients with hereditary transthyretin amyloidosis (ATTRv). The relative abundances of specific genera, especially those belonging to class Clostridia of phylum Firmicutes, were positively correlated with the intraepidermal nerve fiber (IENF) density, and negatively correlated with the severity of small- and larger-fiber neuropathy represented by thermal thresholds on QST and vibratory thresholds on QST and nerve conduction studies in ATTRv patients. Blue: negative correlations; Red: position correlations. *p < 0.05, **p < 0.01, ***p < 0.001.

To understand the clinical significance of altered gut microbiota, we explored the relationship between microbiota profiles and the clinical phenotypes of neuropathy and cardiomyopathy. Intriguingly, the microbiota diversity was associated with cardiac amyloidosis as shown by ^99m^Tc-PYP SPECT imaging, and the patients with a visual score of grade 3 tracer uptake had higher alpha diversity, as shown by the Shannon effective numbers (*p* = 0.033), than those with a visual score of grade 2 (Fig. 4). We further explored the relationships between the bacterial abundance of gut microbiota and the neuropathic parameters of ATTRv, including the NCS Z-score and QSTL Z-score for large-fiber nerves and the IENF density and QSTS Z-score for small-fiber nerves. The abundance of Eubacterium oxidoreducens (Class clostridia) and family XIII AD3011 group (Class clostridia) were inversely correlated with the severity of small-fiber neuropathy measured by the IENF density and thermal thresholds on QST. The abundances of the Lachnospiraceae NK4A136 group (Class clostridia), Oscillospira (Class clostridia), Oscillibacter (Class clostridia), UCG−009 (Class clostridia), UCG−005 (Class clostridia), and Eubacterium oxidoreducens group (Class clostridia) were also inversely correlated with the severity of large-fiber neuropathy assessed by the amplitudes of CMAPs and SNAPs on NCSs and the vibratory thresholds on QST (Fig. 5). There was no correlation between the Firmicutes to Bacteroidetes ratio and the pathophysiological parameters of neuropathy in ATTRv.

## Discussion

In the present study, we compared the gut microbiota taxa of the ATTRv and control groups. All ATTRv patients demonstrated sensorimotor polyneuropathy and cardiomyopathy, as confirmed by pathophysiological studies and radionuclide cardiac imaging. The results showed a distinct alteration of the gut microbiome composition in ATTRv patients with predominant A97S mutations recruited from a Taiwanese population. The relative abundances of some microbiota belonging to Firmicutes, Verrucomicrobia and Actinobacteria were increased, while those of others belonging to Bacteroidetes were decreased in ATTRv. ATTRv patients with a higher degree of myocardial amyloid deposition had an increased richness and diversity of the gut microbiota. Additionally, specific genera of gut microbiota, especially the class Clostridia in the phylum Firmicutes, were associated with parameters of both small- and large-fiber neuropathy in ATTRv patients.

Because the components of the gut microbiota were significantly influenced by age, genetic variation, and diet^21^, a control group for comparison was recruited mainly from the unrelated family members or spouses with similar age to and living-together with the enrolled patients to decrease the confounding effects of these factors on the gut microbiota. The diversity of the fecal microbiota assessed by the ASV richness and Shannon effective numbers of alpha diversity were significantly higher in the ATTRv patients than in the control group, suggesting higher total number and more richness of the ASV in the gut microbiome of ATTRv patients^22,23^. Further comparison of the fecal microbial structure using generalized UniFrac distance showed that the fecal microbiota profiles of the ATTRv patients were distinct from those of the controls, and we identified that the relative abundance of Bacteroidetes was decreased while those of some microorganisms belonging to Firmicutes, Verrucomicrobia and Actinobacteria were increased in ATTRv patients compared to the control group. Additionally, the Firmicutes to Bacteroidetes ratio was much higher in ATTRv patients than in the control group. All these observations strongly suggested that the gut microbiota was altered in patients with ATTRv.

The biodiversity of the microbiota plays an important role in maintaining the stability and functions of the gut ecosystem^24^. Gastrointestinal dysfunction, particularly constipation and diarrhea, is the common symptoms and often appears early during the clinical course of ATTRv^19^. Increasing evidence has documented that gut microbiota dysbiosis is associated with the development of gastrointestinal dysfunction, and changes in gastrointestinal motility may also cause changes in the gut environment and microbiota dysbiosis^25,26^. For example, irritable bowel syndrome is considered to be a disorder of brain-gut interaction, frequently causes diarrhea or constipation and is associated with small intestinal bacterial overgrowth and decreased gut microbiota diversity^27^. Similarly, decreased microbiota diversity is noted in Crohn’s disease, an inflammatory bowel disease that frequently causes diarrhea. In the present study, we did not find significant changes in the diversity of gut mictobiota between ATTRv patients with and without diarrhea or constipation, possibly suggesting additional factors might be related to the altered microbiota in ATTRv in addition to the common gastrointestinal symptoms. However, the number of enrolled patients in the present study might be too small to make a solid conclusion. Additionally small bowel bacterial overgrowth and bile acid malabsorption are also common complications of ATTRv patients^20,28^. In the present study we did not perform direct culture of small intestinal aspirates and breath test to assess the bacterial load of small intestine or ^75^Se-homocholicacid-taurine (SeHCAT) test to evaluate the absorption of bile acid, and whether these complications are associated with the changes of gut microbiota needs further investigations. Another interesting finding is that higher alpha diversity is associated with a higher visual score on ^99m^Tc-PYP SPECT imaging, implying the significance of altered microbiota in cardiac amyloidosis in ATTRv. Previous study has shown significant difference of bacterial components in patients with immunoglobulin light chain amyloidosis compared to controls, and the baseline gut microbiota was associated with disease phenotypes^29^. Certainly, the causal relationships among these findings require further longitudinal study.

In addition to diversity, there was alteration in the composition of the gut microbiota in the ATTRv. In particular, specific bacterial species were associated with distinct nerve fiber type deficits. The relative abundances of Bacteroidetes and Firmicutes were decreased and increased, respectively, and the Firmicutes to Bacteroidetes ratio was markedly increased in the ATTRv group compared to the control group. Additionally, the abundance of some Firmicutes, especially those belonging to class Clostridia, was inversely correlated with the severity of neuropathic parameters, including (1) a higher abundance of the Eubacterium oxidoreducens and family XIII AD3011 group was associated with the intactness of small-fiber pathology and function (higher IENF density in skin biopsy samples and less abnormal thermal thresholds on QST); and (2) a higher abundance of the Lachnospiraceae NK4A136 group, Oscillospira, Oscillibacter, UCG−009, UCG−005, and Eubacterium oxidoreducens group was associated with the integrity of large-fiber neurophysiology and function (higher amplitudes of CMAPs and SNAPs on NCSs and less abnormal vibratory thresholds on QST). These findings are compatible with a recent study in which the firmicutes were negatively correlated the Toronto clinical scoring system in diabetic distal symmetric polyneuropathy^16^. Firmicutes and Bacteroidetes are the two most common phyla in the gut and represent more than 90% of the gut microbiota^24^. Both bacterial phyla can function as integral partners in the human metabolic and immune system, can cause serious life-threatening disease and are thought to be related to various disease states^30^. Many studies exploring the relationships between gut microbiota compositions and diseases have found that compositional variation in Firmicutes and Bacteroidetes is associated with the development of non-neurological disorders such as cancer, cardiovascular disease, diabetes, chronic kidney diseases, autoimmune disorders (e,g., psoriasis and systemic sclerosis) etc., and neuropsychological disorders such as Alzheimer’s disease, stroke, Parkinson’s disease, schizophrenia, and autism spectrum disorder^31–35^. The present study provided another line of evidence on the association between variations in gut Firmicutes and Bacteroidetes and ATTRv. An intriguing finding is the inverse relationship between the relative abundances of class Clostridia bacteria and the severity of polyneuropathy assessed by pathophysiological tools, suggesting the beneficial effects of Clostridia in ATTRv patients. The mechanisms for such relationships are unknown. Clostridia, belonging to the phylum Firmicutes, is a commensal bacterium in the human gut and exerts many salutary effects on intestinal homeostasis. Clostridia populates specific regions at the intestinal mucosa in close relationship with intestinal cells^36^, and this position allows them to continuously crosstalk with gut cells and participate in modulating physiologic, metabolic and immune processes in the gut^37^. Clostridium species have been reported to play a probiotic role primarily by energizing intestinal epithelial cells, strengthening the intestinal barrier and interacting with the immune system to alleviate inflammation^38^. Although amyloid deposits composed of mutant TTR are shared by all ATTRv patients, the pathogenesis of polyneuropathy is still unclear. The presence of proinflammatory markers such as TNF-α and IL-1β in the tissue biopsy samples and the elevated cerebrospinal fluid total protein, which was correlated with the small-fiber nerve pathology in the skin of ATTRv patients, suggested that inflammation might play a role in the progression of neuropathy in ATTRv^10,39^. Further investigation is still awaited to determine whether the anti-inflammation and metabolic function of Clostridia through their distinctive biological activities can potentially have positive effects on polyneuropathy in ATTRv. Previous studies had showed factors that might be associated with the phenotypic heterogeneity and variable penetrance of ATTRv, for example, increased mitochondrial DNA copy number was associated with early onset age^40^; and ATTRv seems predominant and occurs earlier in men than in women in some population^41,42^. Other factors that might affect gene expression and cause phenotype variation included somatic mosaicism^43^, epigenetic modification^44^, and modifier genes^45,46^. Whether these factors are associated with alterations of gut microbiota still needs further large-scale study.

## Limitations

Our study has some limitations. First, all subjects were recruited from the same ethnic group, and nearly all patients were p.A117S genotype. The same genotype and ethnicity in the present study may restrict the generalization of the conclusions to other mutations or ethnicities. Second, there was a significant difference in sex distribution and an unequal range of age distribution between the ATTRv group and the control group which may interfere the comparison of gut microbiota between these two groups. Third, our study is a cross-sectional design, which makes it difficult to confirm the causal directions of the correlational link between ATTRv and gut microbiota profiles. Fourth, this study did not investigate metabolic parameters such as proteomics or metabolomics, which are potential mediators related to alterations in gut microbiota compositions; therefore, we could not identify potential signatures for the phenotypes in individuals with ATTRv. In the future, a longitudinal follow-up study enrolling patients of more genotypes and ethnicities, and considering all possible medical comorbidities and gastrointestinal symptoms coupled with an exploration of omics data will help to clarify the roles of altered gut microbiota in disease development and progression.

## Conclusions

In the present study, we found that the gut microbiota in ATTRv significantly altered not only in the richness and evenness but also in the variability of species compared to the control group, especially in the components of Firmicutes and Bacteroidetes. Furthermore, the present study showed that this microbial diversity was associated with phenotypes of cardiomyopathy. Interestingly, the relative abundance of Clostridia, belonging to the phylum Firmicutes, was significantly correlated with the pathophysiological parameters of both small- and large-fiber nerves, suggesting the potential relationships of the gut microbiota with ATTRv. Further elucidation of the interaction between gut microbial and host immunometabolic responses may lead to a better understanding of the pathogenesis of ATTRv.

## Methods

### Subjects

In this case–control study, we enrolled patients with ATTRv from July 2020 to June 2022 based on the following inclusion criteria: (1) the presence of a transthyretin pathogenic mutation, (2) clinical evidence of sensorimotor or autonomic neuropathic symptoms and pathophysiological evidence of axonal polyneuropathy in skin biopsy or nerve conduction studies, and (3) no monoclonal paraprotein in the serum as determined by immunoelectrophoresis. Patients with alternative causes of polyneuropathy, including diabetes mellitus, autoimmune disorders, renal insufficiency, nutrition insufficiency, toxin exposure, infection or malignancy, were excluded. For the comparison of the gut microbiota, we enrolled biologically unrelated family members of ATTRv patients (for example, the spouse) in a control group.

All participants received clinical assessments, including clinical symptom recordings, neurological examinations and questionnaires. Disability was evaluated according to the familial amyloidotic polyneuropathy (FAP) stage: stage 0, no impairment; stage 1, mild neuropathy with the function of the lower limbs affected but not impaired; stage 2, impaired function of lower limbs with aids to assist walking; and stage 3, wheelchair needed or bedridden. The questionnaires included the World Health Organization Quality of Life Instruments (WHOQOL-BREF) to assess quality of life. Laboratory tests included nerve conduction studies (NCSs), quantitative sensory testing, autonomic function tests, and skin biopsies with quantitation of epidermal innervation for evaluation of neuropathy and ^99m^Tc-PYP SPECT imaging for evaluation of cardiomyopathy.

### Sample collection and DNA extraction

Fecal samples were obtained from both the ATTRv patients and the control group, followed by immediate transportation to our laboratory at a cold storage temperature of 4 °C within 24 h of collection. Subsequently, the fecal samples were aliquoted and stored at -80 °C until microbiome analysis. Fecal genomic DNA was extracted by using the Qiagen PowerFecal DNA Isolation Kit according to the manufacturer’s instructions^13^. After amplification, and purification, libraries were sequenced on an Illumina MiSeq platform (Illumina Inc., CA, USA). A detailed description of the sequencing and analysis was provided in the supplementary methods.

### Skin biopsy and the quantification of epidermal innervation

Skin biopsy was performed with a 3 mm punch needle from the lateral side of the distal leg under local anesthesia. Skin sections of 50 μm thickness were immunostained with antiserum to protein gene product 9.5 (PGP 9.5, Cedarlane Laboratories, Burlington, Ontario, Canada). The reaction product was demonstrated with chromogen SG (Vector Laboratories). PGP 9.5(+) epidermal innervation was quantified throughout the depth of the entire section by examiners blinded to the clinical information following an established protocol^47^. Intraepidermal nerve fiber (IENF) density was derived and expressed as fibers/mm. In the distal leg, normative values from our laboratory (mean ± SD, 5th percentile) for IENF were 11.16 ± 3.70 and 5.88 fibers/mm for subjects aged < 60 years, and 7.64 ± 3.08 and 2.50 fibers/mm for subjects aged ≥ 60 years. The cutoff points of IENF density were 5.88 and 2.50 fibers/mm in these two age groups, respectively^48^.

### Quantitative sensory testing

Quantitative sensory testing (QST) was performed using a Thermal Sensory Analyzer and Vibratory Sensory Analyzer (Medoc Advanced Medical System, Minneapolis, MN) to measure sensory thresholds of warm, cold, and vibratory sensations following an established protocol^49^. Thermal thresholds were recorded at the thenar eminence and foot dorsum according to the algorithm of levels and were expressed as the warm and cold threshold temperatures. Vibratory thresholds were measured at the index finger and lateral malleolar process and were expressed in micrometers. These values were compared with normative values for age, which had previously been documented^47–49^. We defined the large-fiber sensory nerve QST Z-score (QSTL Z-score) as the sum of standard deviations from the mean (Z score) based on the reference values in our laboratory for vibratory thresholds at the finger and ankle and the small-fiber sensory nerve QST Z-score (QSTS Z-score) as the sum of Z scores for the warm and cold thresholds at the thenar and dorsal foot regions.

### Nerve conduction studies

Nerve conduction studies (NCSs) was performed with a Nicolet Viking IV Electromyographer (Madison, WI) following standardized methods recommended by the Consensus Development Conference on Standardized Measures in Diabetic Neuropathy. The studied nerves included the sural, peroneal, tibial, median and ulnar nerves^50^. Abnormal results in NCSs were defined as having reduced amplitudes of compound motor action potentials (CMAPs) or sensory nerve action potentials (SNAPs), prolonged distal latencies, or a slowing of the nerve conduction velocity (NCV)^51^. For the NCS Z-score, we calculated the sum of standard deviations from the mean based on the reference values in our laboratory for five NCS parameters, including distal ulnar CMAP, distal peroneal CMAP, distal tibial CMAP, ulnar SNAP and sural SNAP^8^.

### ^99m^Tc-PYP SPECT imaging

Planar and single-photon emission computed tomography (SPECT) imaging of the chest were performed at 3 h after intravenous injection of 20 mCi of ^99m^technetium pyrophosphate (^99m^Tc-PYP). The protocol and parameters for image acquisition followed the joint guidelines established by The Taiwan Society of Cardiology (TSOC) and the Society of Nuclear Medicine of the Republic of China (SNMROC)^52^, as well as the multisocietal expert consensus recommendation^53,54^. The tracer uptake in the left ventricular myocardium was semiquantitatively assessed by the visual score (VS), which was defined as follows: 0, no uptake; 1, uptake less than the rib; 2, uptake equal to that of the rib; and 3, uptake greater than the rib^52^. A positive scan was defined as a VS ≥ 2, which is strongly suggestive of TTR amyloid cardiomyopathy.

### Bioinformatics and statistical analysis

Numerical variables were expressed as the mean ± SD and were compared with t tests or analysis of variance (ANOVA) if the data followed a Gaussian distribution. If the sample size was small, the numerical variables were compared using the nonparametric test. Fisher’s exact test was used to compare categorical data. The above descriptive analyses were performed using Stata software (StataCorp LP, College Station, TX). The results were considered significant at *p* < 0.05.

In the bioinformatic analyses of gut microbiota, the details are addressed in the supplementary methods. In brief, we used DADA2 and the QIIME 2 pipeline (version 2019.7) for sequencing data processing and grouping the sequences into ASVs. Taxonomic assignment was then performed for representative sequences from each ASV using a QIIME2 naive Bayesian classifier trained on the SILVA 138 99% full-length 16S rRNA gene sequence database. The alpha diversity, which describes the richness and evenness of species within a sample, was measured by ASV richness, Shannon effective numbers and Simpson effective numbers to evaluate microbial diversity. Beta diversity, which describes the compositional differences based on qualitative and quantitative assessments in different samples, was conducted through the principal coordinates analysis using generalized UniFrac distances^55^, and analysis of similarities (ANOSIM) was used to test the difference of microbial communities between groups. LEfSe coupled with LDA was used to identify characteristic taxa in correspondence. To explore correlations among the microbiome, QSTL Z-score, QSTS Z-score, NCS Z-score, and IENF density, Spearman correlation analyses were conducted. Only genera that appeared in at least 4 individuals (10% of the cases) were included in the analysis. Selected taxa with significant correlations were visualized using a heatmap. All analyses were conducted using R statistical software (v3.6.3) and the R package “vegan”^56^.

### Ethics approval and informed consent

This study was approved by the Ethics Committee of National Taiwan University Hospital (201903054RINC). Written informed consent was obtained from all participants before all procedures in the study. This study has been carried out in accordance with the Declaration of Helsinki of the World Medical Association as well.

### Supplementary Information

Supplementary Information.

## Data Availability

The raw sequence reads analyzed in this study are available at the Sequence Read Archive database under the Accession Number PRJNA1035916 (https://www.ncbi.nlm.nih.gov/sra/PRJNA1035916).

## Abbreviations

ATTRv: Hereditary transthyretin amyloidosis
ASV: Amplicon sequence variants
CMAP: Compound motor action potential
FAP: Familial amyloidotic polyneuropathy
IENF: Intraepidermal nerve fiber
LDA: Linear discriminant analysis
NCS: Nerve conduction study
PCoA: Principal coordinate analysis
PERMANOVA: Permutational multivariate analysis of variance
QST: Quantitative sensory testing
SNAP: Sensory nerve action potential
^99m^Tc-PYP: Technetium pyrophosphate
TTR: Transthyretin
UniFrac: Unique fraction distance
VS: Visual score
