# Supplementary material for: Altered gut microbiota in Taiwanese A97S predominant transthyretin amyloidosis with polyneuropathy
Source: Sci Rep. 2024 Mar 14;14:6195. doi: 10.1038/s41598-024-56984-5 (PMC10940600; doi:10.1038/s41598-024-56984-5)
Supplement: Supplementary file 1 — Supplementary Information. [file 41598_2024_56984_MOESM1_ESM.docx]

**Supplementary Information**

**Supplementary Results**

**Supplementary Figure 1.** The distribution of age in patients with hereditary transthyretin amyloidosis (ATTRv) and the control group. There was no difference of age between the ATTRv patients and the controls. Bar = mean

**

**

**Supplementary Figure 2.** The alpha and beta diversity in ATTRv patients with and without treatment, diarrhea or constipation. The alpha diversity was assessed by Amplicon Sequence Variants (ASV) richness, Shannon effective numbers and Simpson effective numbers, and the beta diversity was assessed by Bray‒Curtis dissimilarity. There was no difference in the alpha and beta diversity between ATTRv patients with and without treatment (A), diarrhea (B), and constipation (C).

**
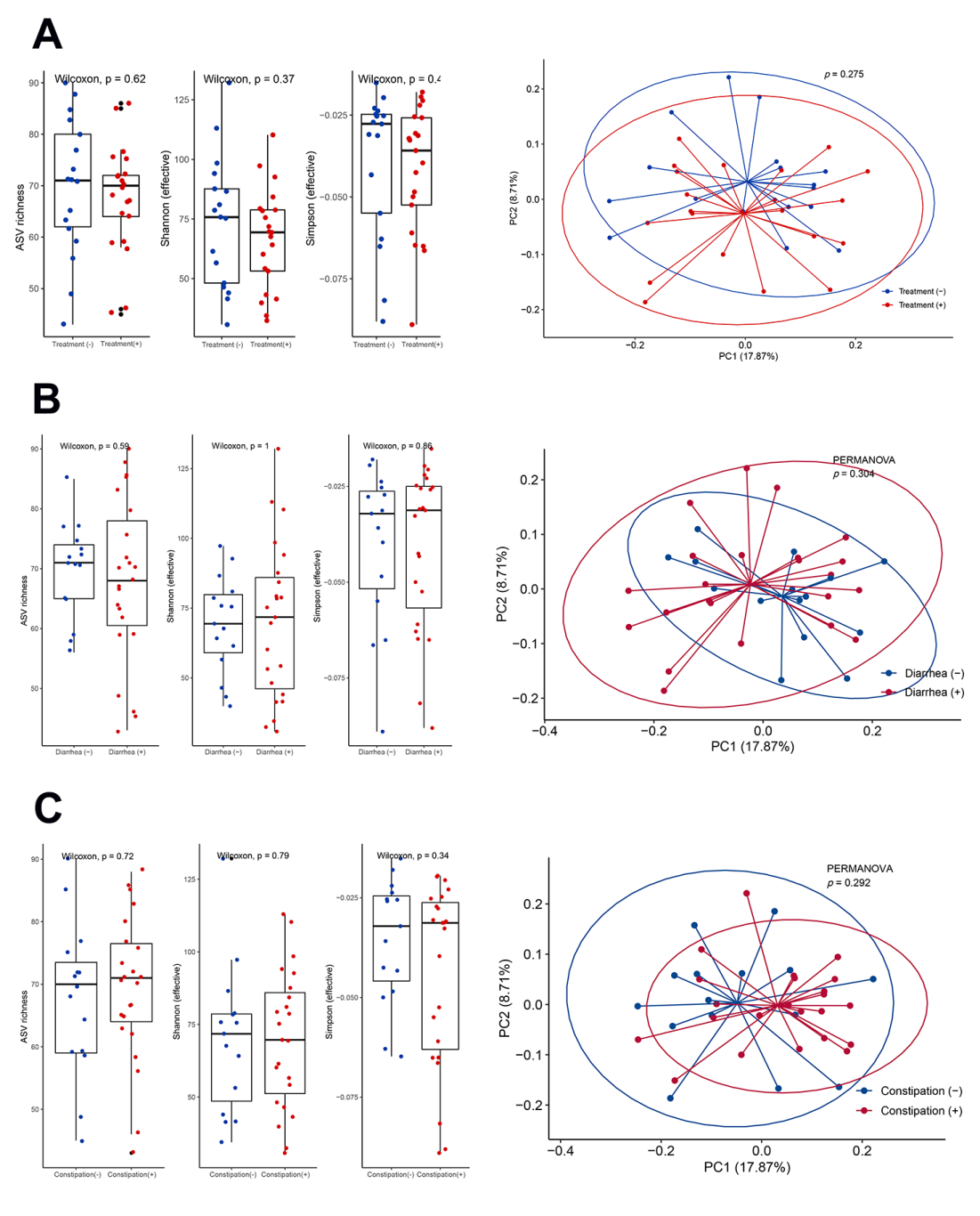
**

**Supplementary methods**

**Gut microbiota composition by 16s rRNA amplicon sequencing**

The integrity and quality of extracted DNA were checked on 1% agarose gel TAE 1X and quantified with a NanoDrop® spectrophotometer. Two steps PCR workflow was performed for library preparation according to the Illumina 16S sample preparation guide. The 16S rRNA gene V3-V4 regions were amplified with a primer overhanging adapter (forward = 5'-TCGTCGGCAGCGTCAGATGTGTATAAGAGACAGCCTA

CGGGNGGCWGCAG-3’; reverse = 5'-TCTCGTGGGCTCGGAGATGTGTATAAG

AGACAGGACTACHVGGGTATCTAATCC-3’). The final libraries (~630 bp) were purified after PCR with AMPure XP beads and were ready for next-generation sequencing. Libraries were denatured and sequenced on an Illumina MiSeq platform to obtain 300-bp paired-end reads and for taxonomic assignment. Detailed descriptions of the amplicons and the sequencing analysis protocol are well mentioned in previous literature^1^.

**Bioinformatics analysis:**

The raw reads were trimmed to remove low quality bases and adapters. The trimmed reads were then processed using DADA2 and the QIIME 2 pipeline (version 2019.7) to perform sequence denoising and the identification of amplicon sequence variants (ASVs)^2^. The ASVs were assigned to taxonomic ranks using a naïve Bayes classifier trained on the SILVA 138 99% full-length 16S rRNA gene sequence database. The relative abundance of each taxon was calculated at different levels, from phylum to genus. The alpha diversity of the samples was measured using three metrics: ASV richness, Shannon effective numbers, and Simpson effective numbers. The ASV richness was calculated as the number of ASVs with relative abundance greater than 0.25% in each sample. The Shannon effective numbers were computed as the exponential of the Shannon entropy index, while the inverse Simpson effective numbers were computed as the reciprocal of the inverse Simpson concentration index^3^. The statistical significance of alpha diversity was evaluated using a Mann–Whitney U test. Beta diversity was assessed through principal coordinate analysis (PCoA) using the generalized UniFrac (unique fraction) distance^4^ after normalization by the total number of reads in that sample. The significance of the beta diversity differences among groups was tested by permutational multivariate analysis of variance (PERMANOVA) using distance matrices.

To identify differences in gut microbiota composition between groups, linear discriminant analysis (LDA) effect size (LEfSe) was employed. Prior to LEfSe analysis, we filtered out the rare taxa that had a mean relative abundance less than 0.1% across all samples. Taxa with an LDA score greater than 3 were considered statistically significant. The LEfSe analysis was performed using the online LEfSe workflow available on the Hutlab Galaxy platform (http://huttenhower.sph.harvard.edu/galaxy/), and the results were visualized in a cladogram based on their phylogenetic relationship.

**References for supplementary information**

1 Chen, C. C. et al. Comparison of DNA stabilizers and storage conditions on preserving fecal microbiota profiles. J Formos Med Assoc **119**, 1791-1798, doi:10.1016/j.jfma.2020.01.013 (2020).

2 Bolyen, E. et al. Reproducible, interactive, scalable and extensible microbiome data science using QIIME 2. Nat Biotechnol **37**, 852-857, doi:10.1038/s41587-019-0209-9 (2019).

3 Jost, L. Partitioning diversity into independent alpha and beta components. Ecology **88**, 2427-2439, doi:10.1890/06-1736.1 (2007).

4 Chen, J. et al. Associating microbiome composition with environmental covariates using generalized UniFrac distances. Bioinformatics **28**, 2106-2113, doi:10.1093/bioinformatics/bts342 (2012).
